# Physician-modified endografts using an endoskeleton ePTFE platform for complex aortic aneurysms requiring a single fenestration: a proof-of-concept study

**DOI:** 10.1186/s42155-026-00753-0

**Published:** 2026-09-09

**Authors:** Kenjiro Kaneko, Takao Ohki, Eisaku Ito

**Affiliations:** 1 Division of Vascular Surgery, Department of Surgery, Shinyurigaoka General Hospital, Kanagawa, Japan; 2 Division of Vascular Surgery, Department of Surgery, The Jikei University School of Medicine, Tokyo, Japan

**Keywords:** Physician-modified endograft (PMEG), Fenestrated endovascular aortic repair, Complex aortic aneurysm, Expanded polytetrafluoroethylene (ePTFE), Juxtarenal aortic aneurysm, Thoracoabdominal aortic aneurysm, Hydrogel coil

## Abstract

**Background:**

Endovascular interventions for complex aortic aneurysms—including juxtarenal abdominal aortic (JAAA), pararenal abdominal aortic (PRAAA), and thoracoabdominal aortic (TAAA) aneurysms—face inherent limitations, such as manufacturing delays and anatomical restrictions. In Japan, where commercial off-the-shelf branched or fenestrated devices remain limited, physician-modified endografts (PMEGs) represent a crucial practical alternative. Conventional polyester-based PMEGs, however, pose challenges regarding manual resheathing and fenestration site fragility. This study evaluated the safety and early- to mid-term clinical outcomes of PMEGs constructed using the endoskeleton-based, expanded polytetrafluoroethylene (ePTFE)-covered AFX VELA platform.

**Methods:**

This retrospective study included four high-risk patients with complex aortic aneurysms requiring a single fenestration. All were unsuitable for open surgical repair (OSR), standard endovascular aneurysm repair (EVAR), or chimney endovascular aneurysm repair (ChEVAR) due to an infrarenal neck length (IRL) < 2 mm. Fenestrations were created via sharp ePTFE incision and reinforced with an expansile hydrogel coil. Following manual resheathing without dedicated crimping tools, target vessel cannulation and bridging stent reconstruction were performed. Endpoints included technical success, 30-day major adverse events (MAEs), target vessel patency, endoleaks, sac diameter changes, and reinterventions.

**Results:**

The cohort (two men, two women; mean age, 73.0 years) presented with saccular aneurysms (one JAAA, two PRAAAs, and one Crawford type V TAAA). The mean maximum diameter was 54.3 mm, and all had an IRL of 0 mm. Technical success was 100%, with zero 30-day mortalities or MAEs. Over a 31.5-month mean follow-up, primary target vessel patency remained 100%, without endoleaks or required reinterventions. Aneurysm sac diameter regressed by 5 mm in two patients and remained stable in two. No aortic-related mortalities occurred.

**Conclusions:**

This proof-of-concept study is the first to describe AFX VELA-based PMEGs and shows that the technique is feasible in carefully selected patients with complex aortic aneurysms. The procedure does not require dedicated crimping tools or complex resheathing maneuvers, whereas its mid- to long-term durability requires continued evaluation. Establishing a definitive clinical role will require larger prospective studies with extended follow-up.

**Graphical Abstract:**

**Figure:**
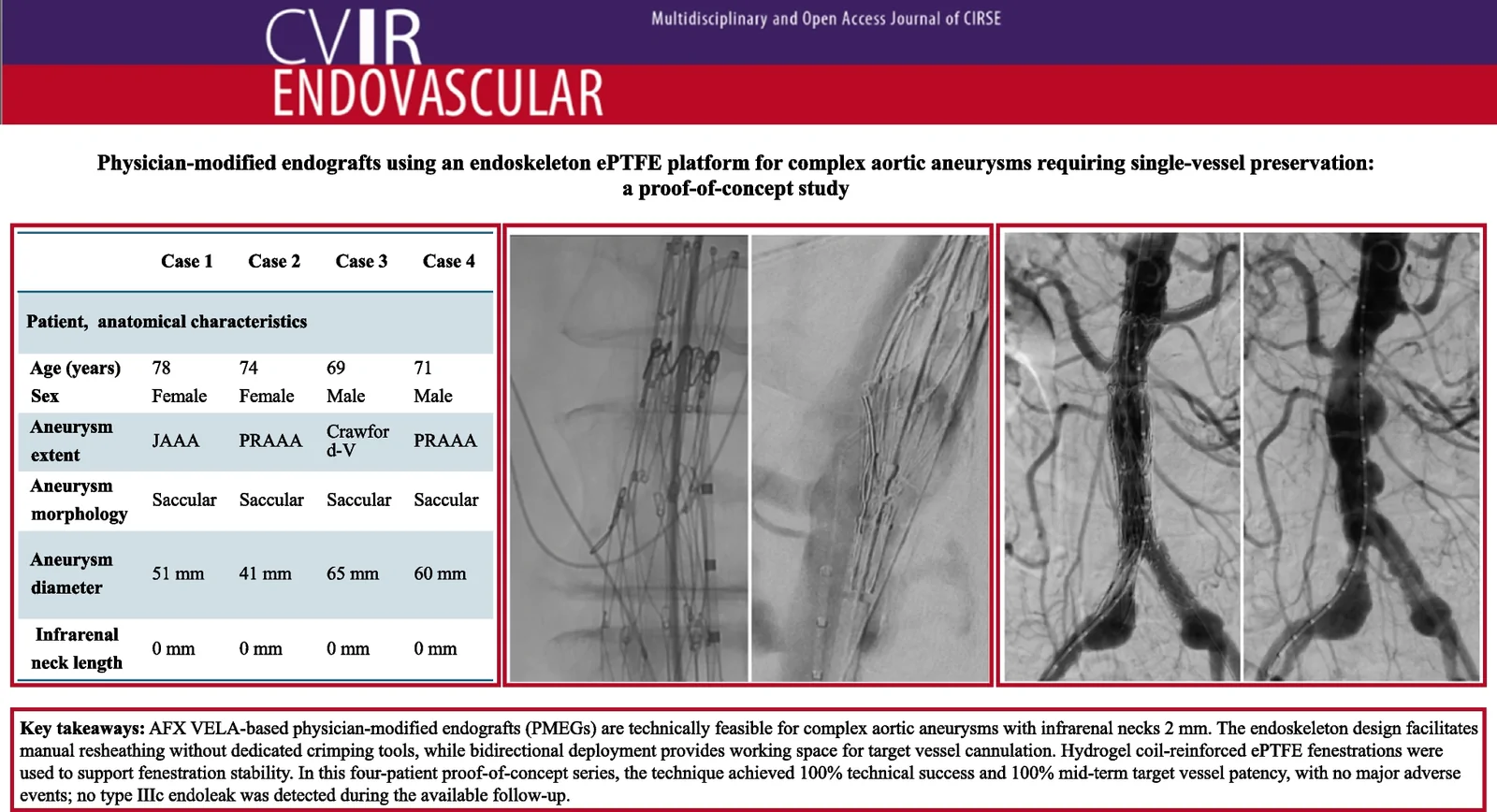

**Supplementary Information:**

The online version contains supplementary material available at https://doi.org/10.1186/s42155-026-00753-0.

## Background

Open surgical repair (OSR) for complex aortic pathologies—encompassing juxtarenal (JAAA), pararenal (PRAAA), and thoracoabdominal aortic aneurysms (TAAA)—remains a highly invasive intervention associated with substantial perioperative morbidity and mortality, particularly in elderly cohorts and patients presenting with severe comorbidities [1–3]. Fenestrated and branched endovascular repair (F/BEVAR) has therefore become a predominant strategy, delivered either as custom-made devices (CMDs) or standardized off-the-shelf devices (OSDs) [1, 4].

Both approaches have practical limits. CMDs require manufacturing that spans several weeks, precluding their use in urgent presentations [5]. OSDs such as the GORE EXCLUDER Thoracoabdominal Branch Endoprosthesis (TAMBE; W. L. Gore & Associates, Flagstaff, AZ, USA) remove this delay but meet anatomical instructions for use in only 23% to 49% of candidates [6]. Furthermore, their use for localized JAAA or PRAAA mandates reconstruction of all four renovisceral branches, adding complexity and aortic coverage disproportionate to the pathology. Chimney EVAR (ChEVAR) requires a minimum infrarenal neck length (IRL) of about 2 mm [7]. In contrast, physician-modified endografts (PMEGs), customized intraoperatively on the back table, are not constrained by IRL and provide immediate availability, with outcomes comparable to CMDs [8, 9].

PMEGs have conventionally used polyester (PET)-based grafts, which pose procedural and structural challenges: resheathing after fenestration is cumbersome and may fail through infolding [10, 11], and fenestration compromises fabric integrity. Although no consensus exists on the long-term superiority of PET versus expanded polytetrafluoroethylene (ePTFE) [12], bench testing shows progressive margin distortion and elastic recoil after fenestration of PET, which may increase the theoretical risk of type III endoleak at the bridging-stent interface [13].

The AFX VELA platform (Endologix, Irvine, CA, USA) combines an internal metallic endoskeleton with an externally mounted ePTFE fabric fixed only at its ends. This unique architecture allows the fabric to be separated from the struts for sharp fenestration and enables manual resheathing without dedicated crimping tools. Its bidirectional deployment permits target vessel cannulation from either direction. Despite these theoretical advantages, clinical evidence for VELA-based PMEGs is lacking. This study evaluated the safety, technical feasibility, and early- to mid-term clinical outcomes of AFX VELA-based PMEGs in patients with complex aortic aneurysms (JAAA, PRAAA, TAAA) requiring a single fenestration who were unsuitable for OSR or standard EVAR.

## Methods

### Study design and patient selection

This single-center, retrospective observational study was conducted in accordance with the Declaration of Helsinki and approved by the Institutional Review Board (Ethics Committee) of our institution (approval number 7–2; July 8, 2015). All patients provided written informed consent for the procedure, including the off-label application of PMEGs. The cohort comprised four consecutive patients who underwent PMEG using the AFX VELA platform. Inclusion required the presence of a JAAA, PRAAA, or TAAA meeting the surgical threshold (saccular aneurysm of any size, or fusiform aneurysm with a maximum short-axis diameter of 50 mm) [1, 14]. Additionally, patients possessed anatomical features unsuitable for conventional ChEVAR, specifically an IRL < 2 mm [7].

In accordance with the 2019 and 2024 European Society for Vascular Surgery (ESVS) guidelines [1, 15] and our previous institutional outcomes [2], OSR was preferred for patients demonstrating a favorable risk profile. Conversely, PMEG was prioritized for individuals exhibiting one or more high-risk criteria for open surgery: (1) a hostile abdomen due to previous major abdominal surgery or radiotherapy; (2) an American Society of Anesthesiologists (ASA) physical status classification of III, or severe systemic comorbidities (e.g., severe chronic obstructive pulmonary disease or depressed cardiac function); (3) tissue fragility secondary to prolonged steroid therapy; (4) concurrent active malignancy; and (5) reduced functional reserve associated with frailty or advanced age (80 years). Patient selection was based on the 2019 ESVS guidelines [1] at the time of treatment; the 2024 revision [15] does not materially change the criteria relevant to this cohort. To ensure the feasibility and safety of this initial experience, we strictly limited the cohort to cases requiring a single fenestration.

#### Endpoints and definitions

Technical success and major adverse events (MAEs) occurring within 30 days postoperatively served as the primary endpoints. We defined technical success as the precise deployment of the planned endograft, successful target vessel cannulation through the fenestration followed by bridging stent placement, absence of type I or III endoleaks on completion angiography, and the avoidance of open conversion [4]. MAEs encompassed any occurrence of all-cause mortality, myocardial infarction, stroke, bowel ischemia, spinal cord ischemia (paraplegia), or renal injury within the 30-day postoperative period. Renal injury was specified as either the initiation of new dialysis or a 50% reduction in estimated glomerular filtration rate (eGFR) from the baseline value [16].

Secondary endpoints evaluated overall survival, aortic-related mortality, primary target vessel patency, endoleak occurrence, longitudinal changes in renal function [17], sac diameter alterations (regression or expansion), and any reinterventions related to the target vessel or aorta. We defined primary target vessel patency as the maintenance of blood flow without occlusion or the need for stenosis-related reinterventions [17]. A meaningful change in sac size (either expansion or regression) required a minimum 5-mm difference in the maximum diameter compared with preoperative measurements [4].

### Preoperative planning and sizing

All patients received a standardized contrast-enhanced computed tomography angiography (CTA) utilizing a slice thickness of 1 mm. We excluded non-dialysis patients with an eGFR of 30 mL/min/1.73 m^2^ and those with contrast medium allergies. Two independent vascular surgeons evaluated the imaging and performed the sizing. They used Ziostation2 software (Ziosoft, Tokyo, Japan) to generate standardized orthogonal centerline reconstructions, evaluating the aneurysm extent, anatomical relationships with branch vessels, and landing zone parameters [18]. Given the saccular morphology of all aneurysms in this cohort, we determined the maximum aneurysm diameter using the greatest dimension encompassing the protruding sac, rather than the standard short-axis measurement on the centerline-orthogonal plane typically applied to fusiform lesions. In compliance with the IFU, we ensured proximal and distal landing-zone lengths of 15 mm for the VELA-based PMEG and established the appropriate oversizing.

### Planning of fenestration sites

Target vessel origins were identified using clock positions along the reconstructed centerline. To determine the circumferential placement of the fenestration, we calculated the arc length on the graft surface based on the target branch angle relative to the centerline axis. Longitudinal coordinates were established by measuring the preoperative CT distance from the upper border of the proximal landing zone (the intended proximal graft edge) to the target vessel origin. This measurement was subsequently replicated on the back table to mark the fenestration site relative to the proximal edge of the VELA graft. Given that the supporting endoskeleton spine of the VELA device naturally aligns with the greater curvature of the aorta, optimal fenestration positioning in these single-branch cases was individualized following careful assessment of each patient’s specific aortic tortuosity. In cases requiring superior mesenteric artery (SMA) fenestration and concomitant coil embolization of the celiac artery, preoperative CT was mandatory to verify adequate collateral circulation between the SMA and celiac artery via the gastroduodenal artery (GDA).

### Sizing of fenestrations and bridging stent grafts

Fenestration diameters were created to be equal to or 1 mm smaller than the baseline target vessel ostium (− 1 to 0 mm). Bridging stent grafts were oversized by 1 mm relative to the target vessel diameter. To optimize the bridging stent pull-out force and mitigate the risk of type IIIc endoleaks [13, 19], intra-aortic flaring (touch-up) was performed using a non-compliant balloon sized 2 mm larger than the target vessel diameter. A balloon-expandable VBX was used as the first-line bridging stent. A self-expanding VIABAHN was reserved for anatomies requiring greater conformability, such as a target vessel arising directly from the aneurysmal sac with a gap between the graft and the vessel ostium.

### Planning of device configuration

For localized lesions, such as saccular aneurysms, the extended overall length of the VELA endograft (95–100 mm) enabled complete aneurysm exclusion using a single device. For more extensive pathology, endograft sizing adhered to standard EVAR protocols, utilizing an appropriate modular configuration that incorporated an AFX main body or equivalent components. Although a VELA suprarenal cuff extension was typically prioritized to minimize the risk of postoperative device migration, a VELA infrarenal cuff extension was selected in the presence of proximal landing zone mural thrombus to mitigate the risk of distal embolization.

### Back-table technique for device modification

**Fig. 1:**
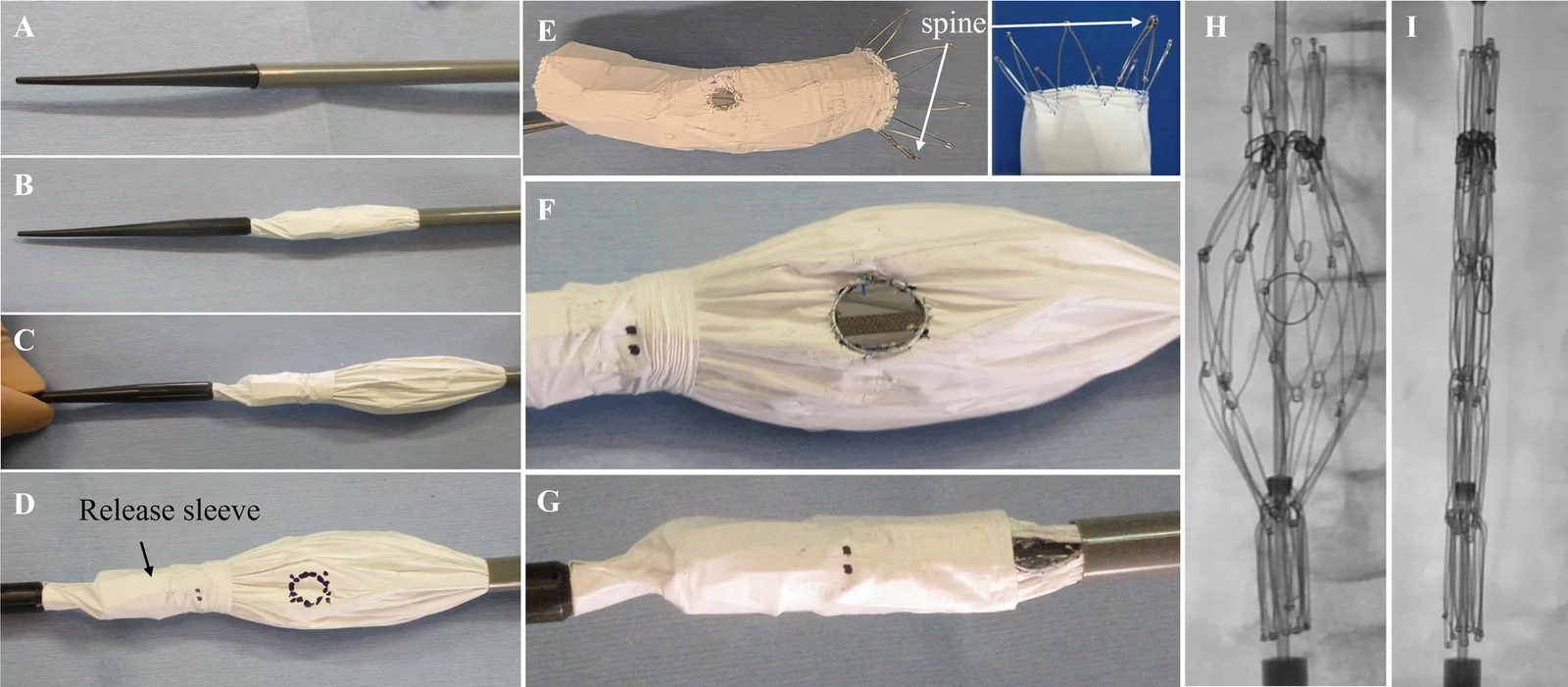
Back-table technique for device modification. **A**–**C** Retracting the outer sheath of the VELA endograft (AFX VELA; Endologix, Irvine, CA, USA) allows for partial deployment of the stent graft up to the intended fenestration level. **D** The proximal stent is identified via palpation or fluoroscopy, subsequently enabling precise marking of the fenestration site based on the measured distance from the proximal graft margin. **E** Given that the supporting endoskeleton spine naturally aligns with the greater curvature of the aorta, the optimal fenestration position is individualized following careful assessment of each patient’s specific aortic tortuosity. **F** An expansile hydrogel coil (AZUR peripheral hydrogel coil system; Terumo, Tokyo, Japan) is circumferentially sutured around the fenestration to provide structural reinforcement. While polypropylene sutures (Prolene; Ethicon, Somerville, NJ, USA) were utilized initially, expanded polytetrafluoroethylene (ePTFE) sutures (CV-5 GORE-TEX Suture; W. L. Gore & Associates, Flagstaff, AZ, USA) are currently preferred to mitigate needle-hole bleeding. **G** Following modification, the release sleeve is repositioned, and the graft is manually resheathed into the outer delivery system without the need for dedicated crimping tools. **H**,** I** Fluoroscopic imaging immediately prior to systemic insertion verifies the structural integrity of the reinforcing coil, confirming that no dislodgement nor deformation has occurred

Intraoperative device modification was performed under strict sterile conditions on the back table. Initially, withdrawing the outer sheath allowed partial deployment of the VELA endograft up to the intended fenestration level (Fig. 1A–C). If the proximal release sleeve obstructed the target area, it was retracted to secure adequate working space. When utilizing a VELA suprarenal cuff extension, we identified the proximal stent by either palpation or fluoroscopy, subsequently marking the precise fenestration site based on the measured distance from the proximal graft margin (Fig. 1D). The supporting-spine side, identified by the paired proximal-stent markers, deploys so as to lie along the greater curvature of the aorta; we therefore used it as a circumferential orientation reference and positioned the fenestration at the clock position of the target vessel relative to this landmark (Fig. 1E).

The unique endoskeleton architecture of the AFX system secures the graft fabric solely at the proximal and distal extremities. Consequently, applying traction separates the fabric from the internal metallic frame, creating a free working area. Because ePTFE precludes thermal cauterization, a 7–0 polypropylene stay suture was applied to provide continuous traction, allowing for precise mechanical excision using a scalpel and Metzenbaum scissors.

For enhanced fluoroscopic visibility, margin protection, and optimized bridging stent apposition, an expansile 0.018-inch hydrogel coil (AZUR peripheral hydrogel coil system; Terumo, Tokyo, Japan) was circumferentially sutured around the orifice. A coil with a loop diameter of either 6 mm or 8 mm and a coil length of 20 cm was selected to match the diameter of each fenestration.

Meticulous care was taken to prevent any coil loops from prolapsing into the graft lumen. The coil was fixed with a continuous suture; in the initial cases a 7–0 polypropylene suture (Prolene; Ethicon, Somerville, NJ, USA) was used (Fig. 1F), and this was later changed to a CV-5 ePTFE suture (GORE-TEX Suture; W. L. Gore & Associates, Flagstaff, AZ, USA). Because the ePTFE suture closely matches its needle track, it reduces suture-line seepage through the needle holes.

Following modification, we restored the release sleeve to its initial position and manually resheathed the stentgraft back into the outer sheath. The endoskeleton design facilitates this manual reloading process without requiring any dedicated crimping instruments (Fig. 1G). Immediately prior to systemic insertion, fluoroscopic imaging verified the structural integrity of the reinforcing coil, ensuring no dislodgement or deformation had occurred (Fig. 1H–I). The step-by-step back-table modification procedure is demonstrated in Additional file 1.

### Modified VELA deployment and cannulation

All procedures were performed under general anesthesia within a hybrid operating room, utilizing percutaneous access via both common femoral arteries. We selected either a retrograde (distal-first) or an antegrade (proximal-first) approach based on individual patient anatomy, specifically evaluating the target vessel angulation and the condition of the access arteries.

**Fig. 2:**
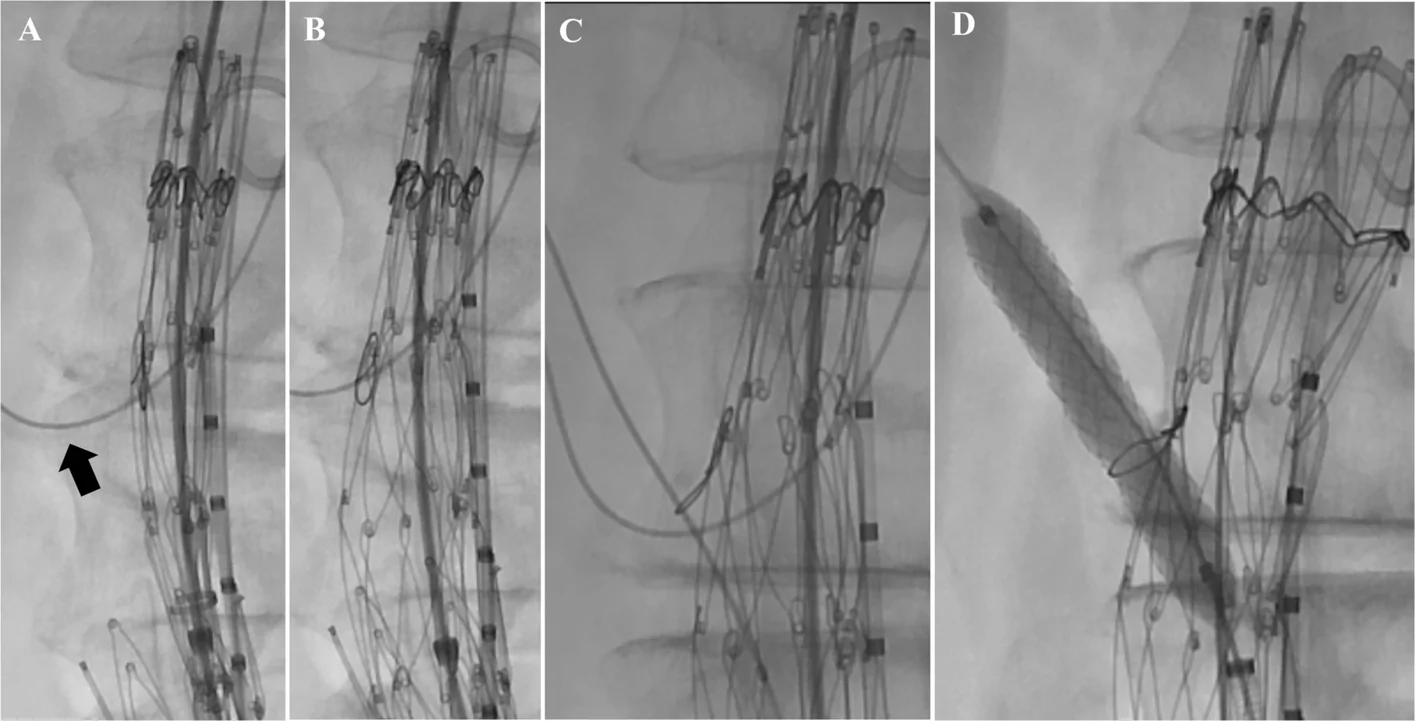
Retrograde approach (Case 1). **A** Preoperative imaging of a juxtarenal abdominal aortic aneurysm. A retrograde cannulation strategy was selected owing to the cranial angulation of the target right renal artery. Because this was the first case, a rescue wire (0.035-inch guidewire) was placed into the right renal artery from the upper extremity before the procedure (arrow); this step was not used in the subsequent cases. **B** Partial deployment of the distal segment, while maintaining proximal constraint (front stop), secures a crucial working space between the endograft and the aortic wall to facilitate target vessel cannulation. **C** Retrograde cannulation of the target vessel is achieved utilizing a 0.035-inch guidewire. **D** Following the advancement of a 100-cm, 7-Fr guiding sheath (Parent Plus; Medikit, Tokyo, Japan) into the target vessel, the proximal portion of the endograft is fully deployed. Subsequently, the bridging stent is introduced, deployed, and flared to complete the reconstruction

**Fig. 3:**
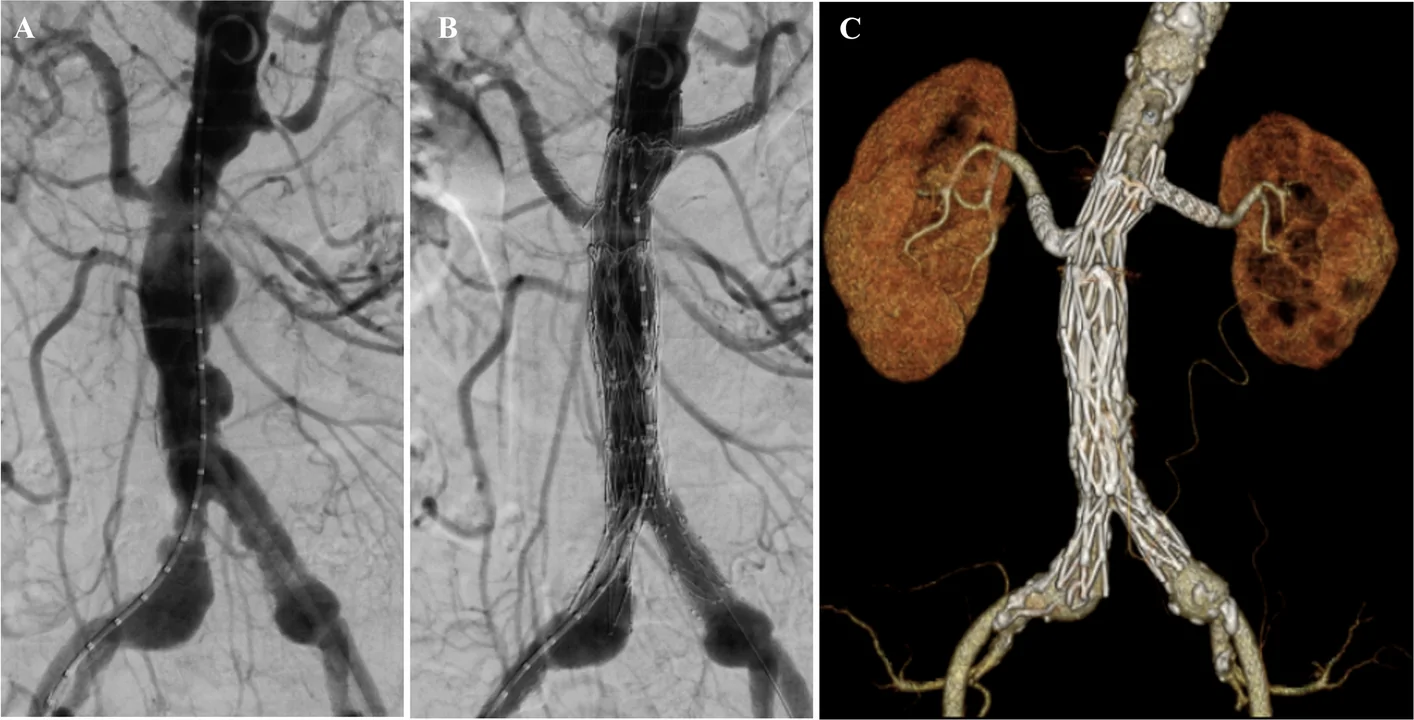
Intraoperative angiography and postoperative three-dimensional computed tomography angiography (CTA) (Case 1). **A** Initial intraoperative angiogram demonstrating the target pathology. **B** Completion angiogram confirming successful aneurysm exclusion and unimpeded flow through the target vessel, without evidence of endoleaks. **C** Postoperative three-dimensional CTA reconstruction demonstrating stable endograft conformation, a patent bridging stent, and complete exclusion of the aneurysm sac

During the single retrograde case, an AFX main body was initially deployed as needed. Subsequently, the modified VELA device was introduced via the ipsilateral approach, while a 100-cm, 7-Fr guiding sheath (Parent Plus; Medikit, Tokyo, Japan) was advanced contralaterally. After positioning the VELA at the optimal level, we withdrew the outer sheath under fluoroscopic guidance—precisely aligning the target vessel with the coil marker—to achieve full deployment of the distal segment (Fig. 2). The release sleeve maintained the proximal portion in a constrained state while the target vessel was cannulated using an appropriate catheter via the contralateral guiding sheath. Following sheath advancement into the target vessel, the release sleeve was opened to fully deploy and anchor the proximal aspect of the VELA endograft (Fig. 3 illustrates the initial intraoperative angiogram, the completion angiogram, and the postoperative three-dimensional CTA (3D-CTA) for this case).

**Fig. 4:**
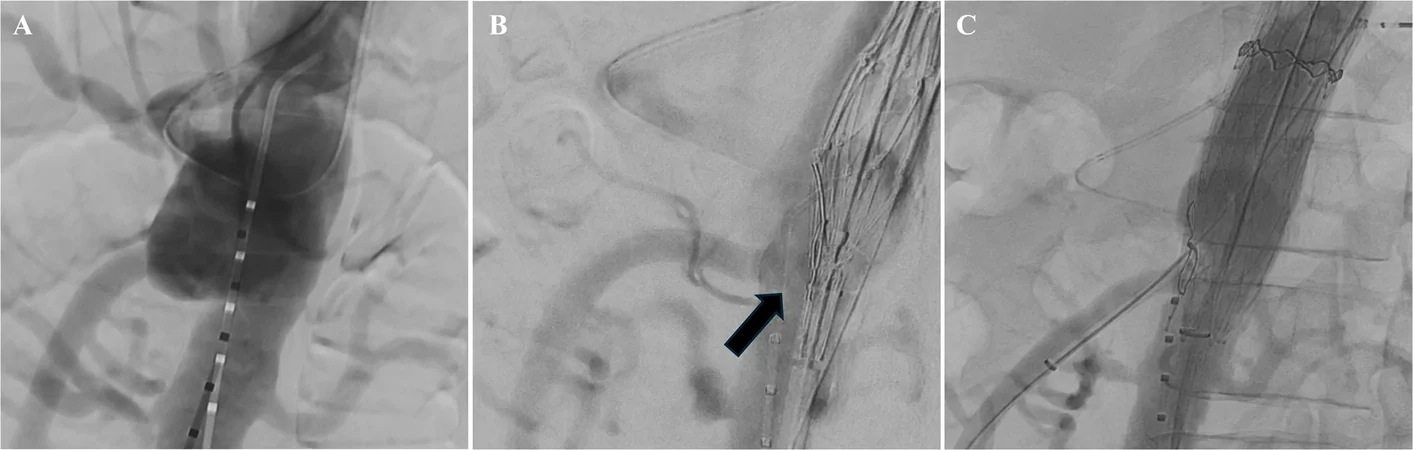
Antegrade approach (Case 3). **A** Preoperative imaging of a Crawford type V thoracoabdominal aortic aneurysm. The distance from the distal aneurysm margin to the left and right renal arteries was 15 mm and 20 mm, respectively. The superior mesenteric artery (SMA) was designated as the target vessel for fenestration, necessitating concomitant celiac artery embolization. Adequate collateral perfusion via the gastroduodenal artery (GDA)—initially evaluated on preoperative computed tomography—was intraoperatively verified by SMA angiography during temporary balloon occlusion of the celiac artery. An antegrade access strategy was selected because of the acute distal angulation of the SMA, following confirmation of minimal thoracic aortic mural thrombus. **B** Following optimal positioning, the front stop is released, and the release sleeve is opened to fully deploy the proximal segment. Maintaining distal constraint (back stop) secures a critical working space between the fenestrated endograft, the aortic wall, and the target branch, thereby facilitating antegrade cannulation (arrow). **C** Analogous to the retrograde approach, a 100-cm, 7-Fr guiding sheath (Parent Plus; Medikit) is advanced into the target vessel. The distal segment of the endograft is then fully deployed, followed by the introduction, deployment, and flaring of the bridging stent

For the three cases in which we used an antegrade approach, we supplemented the bilateral femoral access with a 7-Fr guiding sheath (Parent Plus) inserted via the right brachial artery. Once the VELA device reached the target location, we removed the front stop to initiate deployment and subsequently opened the release sleeve to fully expand the proximal segment (Fig. 4A, B). To facilitate target vessel cannulation, the distal portion (back stop) remained intentionally constrained. Subsequent procedural steps mirrored those of the retrograde approach: the guiding sheath was advanced into the target branch (Fig. 4C), followed by full distal deployment and introduction and flaring of the bridging stent. Throughout these steps—particularly with the antegrade approach, in which the closed distal end of the VELA is exposed to antegrade blood flow—an assistant manually held the main delivery device to prevent flow-induced migration, and device position was confirmed against the radiopaque markers under fluoroscopy. To prevent inadvertent wire passage into the space between the endoskeleton and the graft fabric, an angled catheter was advanced and rotated to confirm that its tip had not entered this space; the tip was then aligned with the fenestration before wire insertion. In cases requiring SMA fenestration, intraoperative verification of collateral flow to the celiac artery via the GDA—initially observed on preoperative CT—was mandatory. Following transient balloon occlusion of the celiac artery, selective SMA angiography confirmed adequate hepatic and splenic perfusion. This validated the use of a single SMA fenestration, and we subsequently performed coil embolization of the celiac artery. In this patient, who was on maintenance hemodialysis with negligible residual urine output, preservation of renal perfusion was not required; both main renal arteries were therefore intentionally covered without reconstruction. Prophylactic coil embolization of the renal arteries was not performed, as the conformable ePTFE sealing of the VELA cuff against the aortic wall was expected to limit persistent retrograde flow into the excluded segment.

### Follow-up protocol

Adhering to our institutional surveillance protocol, postoperative follow-up comprised clinical evaluations, blood chemistry analyses, and CTA at 1, 6, and 12 months, followed by semi-annual or annual assessments thereafter. In patients with baseline renal impairment precluding contrast administration, alternative imaging consisted of non-contrast CT to evaluate aneurysm sac diameter and device integrity, combined with duplex ultrasonography to assess target vessel patency.

### Statistical analysis

Because of the limited size of this proof-of-concept cohort (*n* = 4), we did not perform formal statistical hypothesis testing. The analysis is strictly descriptive; continuous variables are expressed as means accompanied by observed ranges, whereas categorical variables are presented as frequencies (counts).

## Results

### Patient and anatomical characteristics

Table 1 summarizes the baseline demographics and anatomical features of the four consecutive patients. The cohort consisted of two men and two women, with ages ranging from 69 to 78 years (mean, 73.0 years) and an ASA physical status classification of I to III. All individuals fulfilled the institutional criteria for high-risk OSR owing to various severe comorbidities (Table 1). Morphologically, all aneurysms were saccular, comprising one JAAA, two PRAAAs, and one Crawford type V TAAA. The maximum aneurysm diameter ranged from 41 to 65 mm (mean, 54.3 mm). Furthermore, an IRL of 0 mm was present in all cases, rendering the entire cohort anatomically unsuitable for standard EVAR or ChEVAR.

**Table 1:** Patient demographics and anatomical characteristics

Variable	Case 1	Case 2	Case 3	Case 4
Patient demographics				
Age (years)/sex	78/female	74/female	69/male	71/male
ASA physical status classification	II	I	II	III
Main high-risk factors for open repair	Severe COPD, Steroid use	Active chemotherapy, Steroid use	Post-stroke frailty	Decompensated heart failure, HD
eGFR (mL/min/1.73 m^2^)	53	58	37	9
Anatomical characteristics				
Aneurysm extent	JAAA	PRAAA	Crawford-V	PRAAA
Aneurysm morphology	Saccular	Saccular	Saccular	Saccular
Maximum aneurysm diameter (mm)	51	41	65	60
Infrarenal neck length: IRL (mm)	0	0	0	0

Data are presented individually for each of the four patients. Maximum aneurysm diameter refers to the greatest sac dimension; all aneurysms were saccular. IRL denotes the length of the non-aneurysmal aortic segment between the lowest renal artery and the proximal margin of the aneurysm. Crawford-V refers to the Crawford classification for thoracoabdominal aortic aneurysm. No statistical comparison was performed given the small sample size (*n* = 4, proof-of-concept)

*Abbreviations*: *ASA* American Society of Anesthesiologists, *COPD* chronic obstructive pulmonary disease, *eGFR* estimated glomerular filtration rate, *HD *hemodialysis, *IRL* infrarenal neck length, *JAAA* juxtarenal abdominal aortic aneurysm, *PRAAA* pararenal abdominal aortic aneurysm

### Procedural details and device configuration

Table 2 details the procedural variables and device configurations. The targeted branch for the PMEG was the right renal artery in two patients and the SMA in the other two. Endograft deployment proceeded via an antegrade (proximal-first) approach in three cases and a retrograde (distal-first) approach in one. The back-table device modification—comprising fenestration creation, hydrogel-coil reinforcement, and resheathing of the endograft—required 15 to 25 min (mean, 18.3 min). Complete aneurysm exclusion was achieved utilizing the VELA device alone in two instances; the other two cases necessitated adjunctive components (an AFX main body or an aorto-uni-iliac device) tailored to specific anatomical requirements. For the bridging stent, a balloon-expandable GORE VIABAHN VBX Endoprosthesis (W. L. Gore & Associates) was utilized in three patients and a self-expanding VIABAHN endoprosthesis (W. L. Gore & Associates) in one, selected based on individual target vessel anatomy. Anatomically dictated adjunctive procedures included coil embolization of the celiac artery (two cases), ChEVAR for the contralateral renal artery (one case), and the deployment of a bare-metal stent distal to the bridging stent (one case). Operative metrics demonstrated a total procedure time of 203 to 243 min (mean, 224.3 min), fluoroscopy time of 43.0 to 63.5 min (mean, 57.4 min), contrast medium volume of 284 to 418 mL (mean, 343.0 mL), and estimated blood loss of 250 to 430 mL (mean, 317.0 mL).

**Table 2:** Operative details

Operative details	Case 1	Case 2	Case 3	Case 4
PMEG target vessel	Right RA	Right RA	SMA	SMA
Target vessel approach	Retrograde	Antegrade	Antegrade	Antegrade
Graft modification time on back-table (min)	25	18	15	15
Aortic device configuration	Main body and VELA (Suprarenal)	VELA (Infrarenal) only	VELA (Suprarenal) only	Endurant AUI and VELA (Suprarenal)
Bridging stent	VBX 7–39	VIABAHN 6–50	VBX 8–39	VBX 8–39
Adjunctive procedures	VBX 7 × 39 mm in the left renal artery^a^ Additional VELA (infrarenal) deployment^b^	VIABAHN 5 × 50 mm chimney stent in the left renal artery^c^ SMART 6 × 60 mm stent in the right renal artery^d^	Celiac artery coil embolization^e^	Endurant II + Gore Excluder leg^f^ Celiac artery coil embolization^e^
Total operative time (min)	203	243	217	234
Fluoroscopy time (min)	60.4	63.5	43.0	62.5
Contrast volume (mL)	284	320	418	350
Estimated blood loss (mL)	338	250	430	250

^a^A VBX 7 × 39 mm balloon-expandable stent graft (GORE VIABAHN VBX; W. L. Gore & Associates, Flagstaff, AZ, USA) was placed in the left renal artery because of severe (99%) ostial stenosis

^b^Because the overlap between the main body and the VELA was short (1 cm), an additional VELA (infrarenal) was deployed to prevent type IIIa endoleak

^c^The right renal artery was incorporated through the fenestration of the PMEG, with the proximal end of the VELA (infrarenal) positioned immediately below the SMA. For the left renal artery, because the distance from its ostium to the proximal margin of the aneurysm was short (6 mm), chimney EVAR was performed, and a VIABAHN 5 × 50 mm endoprosthesis (GORE VIABAHN; W. L. Gore & Associates) was placed as the chimney stent

^d^The VIABAHN 6 × 50 mm used as the bridging stent for the right renal artery expanded incompletely; a SMART 6 × 60 mm stent (Cordis S.M.A.R.T.; Cordis, Miami Lakes, FL, USA) was therefore added across the affected segment to secure flow

^e^The celiac artery was first occluded with a balloon, and angiography from within the SMA confirmed adequate hepatic and splenic perfusion through the gastroduodenal artery. After a single fenestration (SMA) PMEG, the celiac artery was embolized with coils through a pre-positioned catheter

^f^This patient had previously undergone femoro-femoral bypass for right iliac occlusion at another institution. Treatment was performed through left access; because the left internal iliac artery was occluded, the device was landed in the external iliac artery. After deployment of the Endurant II Aorto-Uni-Iliac (AUI) Stent Graft System (Medtronic, Minneapolis, MN, USA), a modified VELA was deployed proximally and a GORE Excluder leg (W. L. Gore & Associates) was added distally

### Primary endpoints

Table 3 presents the primary endpoint outcomes. Technical success was achieved in all cases (100%). This was characterized by accurate deployment of the intended device, successful target vessel cannulation and bridging stent placement via the fenestration, absence of type I or III endoleaks on completion angiography, and no requirement for open surgical conversion. During the 30-day postoperative period, no MAEs occurred; the mortality rate was 0%, and no patients experienced myocardial infarction, stroke, bowel ischemia, spinal cord ischemia (paraplegia), or renal injury (defined as a 50% reduction in baseline eGFR or the initiation of new dialysis). The length of hospital stay ranged from 4 to 6 days (mean, 5.3 days), with all patients ambulating independently upon discharge to their homes.

**Table 3:** Primary and secondary endpoint outcomes

Endpoints	Case 1	Case 2	Case 3	Case 4
Primary				
Technical success	Yes	Yes	Yes	Yes
Endoleak at completion	None	None	None	None
Open conversion	None	None	None	None
Perioperative mortality	None	None	None	None
Perioperative major adverse events	None	None	None	None
eGFR at 1 month (mL/min/1.73 m^2^)	56	55	30	9
Length of hospital stay (days)	6	6	5	4
Secondary				
Overall survival	Alive	Dead (MDS)	Alive	Alive
Aortic-related mortality	None	None	None	None
Primary target vessel patency	Patent	Patent	Patent	Patent
Endoleak	None	None	None	None
Sac behavior at latest CT	Regression (42 mm)	Stable (39 mm)	Regression (56 mm)	Stable (58 mm)
Aortic events/reintervention	None	None	None	None
eGFR at final examination (mL/min/1.73 m^2^)	58	57	35	8
Follow-up duration (months)	49	40	17	20

Definitions

Technical success was defined as successful deployment of the intended device with target vessel cannulation, without type I or III endoleak or unplanned open conversion

Major adverse events comprised all-cause death, myocardial infarction, stroke, bowel ischemia, spinal cord ischemia, and renal injury (a 50% decrease in eGFR from baseline or the initiation of new dialysis) within 30 days

Primary target vessel patency denotes maintained flow without target vessel occlusion or reintervention for stenosis

Sac regression and expansion were defined as a change of ≥ 5 mm in maximum sac diameter compared with the preoperative measurement; values in parentheses indicate the maximum diameter at the latest computed tomography

In Case 2, the latest imaging follow-up was at 40 months; the patient subsequently died of MDS at 41 months, unrelated to the aorta

*Abbreviations*: *CT* computed tomography, *eGFR* estimated glomerular filtration rate, *MDS* myelodysplastic syndrome

### Secondary endpoints

Table 3 also delineates the secondary endpoint outcomes. The follow-up period ranged from 17 to 49 months (mean, 31.5 months). Although one patient succumbed to myelodysplastic syndrome at 41 months postoperatively, the cohort experienced zero aortic-related mortalities. Throughout the follow-up duration, primary patency of the target vessel was 100% (4/4 branches). Surveillance imaging, comprising CT and duplex ultrasonography, revealed complete absence of endoleaks, including any type IIIa or IIIc endoleaks originating from the fenestration or modular junctions. Consequently, no reinterventions related to the aorta or target vessels were necessary. Postoperative renal function remained stable, with no clinically relevant decline in eGFR from baseline and no need for new hemodialysis (Tables 1 and 3). At the latest CT evaluation, sac regression (**≥ **5 mm reduction in diameter) was evident in two aneurysms, while the remaining two maintained stable dimensions; notably, no aneurysm expansion was observed.

## Discussion

To our knowledge, this is the first clinical report of PMEGs constructed from the AFX VELA platform. In four consecutive patients with complex aortic aneurysms and an infrarenal neck unsuitable for OSR or standard endovascular repair, the procedure was completed as intended in every case. During early- to mid-term follow-up, no fenestration or bridging stent-related endoleak was detected, and target vessel patency was preserved without bridging stent occlusion. These findings indicate that the endoskeleton ePTFE construction can be adapted for physician modification with acceptable mid-term stability, and suggest that the technique is feasible in carefully selected patients whose anatomy precludes established options. The discussion below examines how the specific properties of this platform address the principal limitations of conventional PMEG.

Currently, PMEG serves as a widely embraced endovascular strategy for managing complex aortic aneurysms. A large multicenter international registry encompassing 1274 cases demonstrated a 94% technical success rate alongside a 5.8% 30-day mortality rate [20]. Similarly, a single-center investigational device exemption trial involving 203 patients documented technical success and early mortality rates of 93.7% and 2.9%, respectively [5]. Our described method falls within this spectrum of PMEG interventions. Given the consistently high technical success rates, PMEG is rapidly solidifying its role as a practical alternative for anatomies that are unsuitable for standard EVAR or open surgery. Nevertheless, large PMEG series report type Ic/IIIc endoleaks—originating from the fenestration or the bridging stent interface—in approximately 3% to 10% of cases [4, 5, 21], indicating that the long-term durability of these created fenestrations remains an unresolved issue. Additionally, the obligatory resheathing of the modified graft following back-table fenestration introduces significant procedural complexity, with the literature describing deployment failures directly linked to this specific maneuver [22]. Such challenges are ubiquitous across conventional PMEG procedures and fundamentally stem from the inherent device architecture combined with the mechanical nature of the fenestration technique. Our approach aims to overcome these limitations by utilizing the VELA platform. Its unique endoskeleton construction separates the ePTFE fabric from the internal metallic frame, which not only allows for fenestration independent of the metal struts but also enables manual resheathing without the need for dedicated crimping tools.

In conventional PMEG procedures, reloading the modified endograft constitutes a significant rate-limiting step. Chan et al. previously documented that resheathing a 36-mm main body into a 20-Fr delivery system was markedly challenging; the partial infolding of the graft presumed to occur during this maneuver ultimately resulted in failed renal cannulation, a type III endoleak, and subsequent conversion to open surgical repair [22]. This underscores how the inherent difficulty of the resheathing process can directly precipitate deployment failure. The endoskeleton design of the VELA platform prevents direct compression of the metallic frame against the delivery sheath, thereby facilitating manual resheathing without the need for dedicated crimping devices. Although reloading the release-sleeve segment occasionally proved demanding and required operator familiarity, successful deployment without any evidence of infolding was achieved across all four cases in our cohort.

Furthermore, the unique bidirectional deployment mechanism of the VELA device offers versatility in selecting the target vessel cannulation route. Initial deployment of the proximal segment while maintaining distal constraint secures a crucial working space between the fenestrated endograft, the aortic wall, and the target branch, facilitating antegrade cannulation from an upper extremity approach; a similar principle applies to the retrograde approach. Consequently, we individualized the access strategy based on patient anatomy, utilizing a retrograde approach from the distal side in one instance and an antegrade approach via the upper extremity in three. We favored an antegrade strategy when the target vessel exhibited acute distal angulation in the absence of substantial thoracic aortic mural thrombus. Conversely, a retrograde approach was preferred for cranially directed branches or in the presence of severe thoracic mural thrombus (shaggy aorta). This procedural adaptability is particularly beneficial for cannulating renal and superior mesenteric arteries, which frequently present with acute branching angles.

Regarding graft materials, PET has traditionally been the preferred fabric for PMEG construction across most centers. This preference partially stems from a fabrication constraint: whereas PET allows for fenestration margin reinforcement via thermal cautery, ePTFE is generally unsuitable for heat sealing [5]. Although the VELA platform utilizes an ePTFE covering, we created fenestrations using sharp mechanical incisions rather than thermal cautery, an approach that appears advantageous for preventing unintended and extensive thermal damage to the graft fabric. An in vitro analysis by Eadie et al. evaluated the impact of fenestration on various graft fabrics, subjecting three PET products and two ePTFE grafts to radiofrequency puncture and balloon dilation followed by accelerated fatigue testing. Post-fatigue evaluation revealed that significant fenestration area expansion was restricted exclusively to a monofilament-woven PET product (Talent; increasing from 6.25 to 8.17 mm^2^, *p* = 0.006), with no significant dimensional alterations observed in the other PET or ePTFE grafts. The investigators concluded that device-specific textile architecture, rather than the broad material categorization of PET versus ePTFE, primarily determines post-fenestration mechanical behavior, while also noting the considerable variability in the mechanical properties of ePTFE [23].

In PMEG procedures, the long-term stability of the fenestration remains a principal concern, as progressive deformation of the margin or component separation at the bridging stent interface may precipitate type IIIc endoleaks. To address this, our technique incorporated an expansile hydrogel coil sutured along the entire fenestration circumference, aiming to protect the incised edge and optimize bridging stent apposition. Iwakoshi et al. previously described a comparable reinforcement strategy utilizing an AFX-based platform within the iliac segment. For 15 patients deemed anatomically unsuitable for commercial iliac branch devices, the authors successfully preserved internal iliac perfusion using a hydrogel coil-reinforced fenestration in an AFX limb. They reported a 100% technical success rate, with zero branch occlusions or type IIIc endoleaks during a mean follow-up of 11.6 months [24]. Despite differences in hemodynamics and branch angulations between the internal iliac artery and the renovisceral segment, their findings suggest that combining a hydrogel coil-reinforced fenestration with the AFX endoskeleton architecture yields stable mid-term outcomes across different vascular beds. In our four-patient cohort, primary target vessel patency was maintained throughout follow-up, with no fenestration-related endoleak or aortic-related reintervention. These observations are consistent with adequate short- to mid-term behavior of the hydrogel coil-reinforced fenestration on the ePTFE-covered VELA platform. They do not, however, establish the long-term integrity of the fenestration margin. Repetitive pulsatile loading at the incised edge and at the bridging stent interface could still lead to gradual margin deformation or component separation over time, and the present follow-up is too short to exclude these modes of late failure. The durability of the fenestration margin therefore remains uncertain and warrants continued long-term surveillance.

The optimal bridging stent for PMEG fenestrations has not been established, and both balloon-expandable and self-expanding covered stents have been used according to target vessel anatomy and operator preference [22]. In the present cohort, a self-expanding VIABAHN was chosen for the right renal artery in Case 2. Here the target vessel arose directly from the sac of the PRAAA, leaving a gap between the VELA graft and the vessel ostium; a more conformable self-expanding stent was therefore preferred to bridge this gap. Although a self-expanding stent cannot be flared to the same degree as a balloon-expandable device, potentially increasing the theoretical risk of type IIIc endoleak, the circumferential hydrogel coil reinforcing the fenestration was considered to mitigate this risk. Because standardized off-the-shelf platforms such as TAMBE employ the VBX as their dedicated bridging component [25], our current institutional policy favors the VBX as the first-line bridging stent, reserving the self-expanding VIABAHN for anatomies demanding greater conformability.

Although the off-the-shelf TAMBE device recently became available in Japan, a recent analysis demonstrated no significant differences in mid-term outcomes between TAMBE and PMEGs [21]. Furthermore, our modified VELA technique provides a crucial endovascular option for short-neck anatomies where off-the-shelf devices are anatomically contraindicated, particularly in patients with an IRL of < 2 mm [7]. Notably, current Japanese guidelines do not yet address PMEGs [14]. Therefore, while this technique was performed strictly as an off-label procedure under rigorous institutional oversight, its formal clinical role necessitates ongoing evaluation.

This study is subject to several limitations. First, the retrospective, single-center design comprising only four consecutive patients without a comparative control group inherently precluded the application of formal statistical analyses. Second, the specific configuration combining the VELA endograft, the mechanical fenestration technique, and bridging stents from alternative manufacturers constitutes an entirely off-label application; thus, the long-term safety of this structural construct remains unverified. Third, to prioritize patient safety during this initial experience, we deliberately restricted the cohort to patients requiring a single fenestration, and all treated aneurysms were saccular. Extending the technique to multiple fenestrations warrants comment. Creating a fenestration within the segment covered by the proximal release sleeve, where reloading is inherently more demanding, may increase the difficulty of manual resheathing when several fenestrations are combined. However, manual resheathing of a graft with a single fenestration met almost no resistance in our experience, which suggests that adding fenestrations outside the release-sleeve segment may be feasible; this possibility nonetheless requires confirmation in bench testing and further clinical use. The applicability of the technique to fusiform aneurysms is likewise unproven. Fusiform morphology generally demands a longer treatment length and additional modular components, and adequate modular overlap is particularly important with the AFX platform to avoid type IIIa endoleak. The resulting need for longer coverage and greater overlap may constrain the use of AFX-based PMEGs in extensive fusiform disease, although the addition of a cuff between the VELA and the main body may compensate for this, provided that the anatomy of each case is fully characterized.

Finally, the mid-term follow-up duration is insufficient to comprehensively evaluate the long-term durability of the PMEG, particularly regarding the structural integrity of the fenestration margin and the bridging stent interface. Consequently, while this report successfully demonstrates technical feasibility and favorable early- to mid-term outcomes, prospective studies incorporating larger patient cohorts and extended follow-up are essential to firmly establish the definitive clinical utility of this technique.

## Conclusions

This proof-of-concept study is the first to describe AFX VELA-based PMEGs and shows that the technique is feasible in carefully selected patients with complex aortic aneurysms. In four patients with an infrarenal neck length of < 2 mm, the procedure achieved 100% technical success, with mid-term target vessel patency and stable aneurysm exclusion. The endoskeleton architecture and bidirectional deployment of the VELA device facilitated manual resheathing and target vessel cannulation without dedicated crimping tools or complex resheathing maneuvers. Combining sharp ePTFE incision with hydrogel coil reinforcement supported fenestration stability, and no type IIIc endoleak was detected during follow-up. This preliminary, single-center experience requires larger prospective studies with extended follow-up before the clinical role of VELA-based PMEGs can be defined.

## Supplementary Information

Additional file 1. Video demonstrating the back-table device modification technique. The procedure includes partial deployment of the AFX VELA endograft, precise mechanical fenestration of the ePTFE fabric, circumferential reinforcement using an expansile hydrogel coil, and effortless manual resheathing without dedicated crimping tools.

## Data Availability

The datasets used and/or analyzed during the current study are available from the corresponding author on reasonable request.

## Abbreviations

ASA: American Society of Anesthesiologists
ChEVAR: Chimney endovascular aneurysm repair
CMD: Custom-made device
CTA: Computed tomography angiography
eGFR: Estimated glomerular filtration rate
ePTFE: Expanded polytetrafluoroethylene
ESVS: European Society for Vascular Surgery
EVAR: Endovascular aneurysm repair
F/BEVAR: Fenestrated and branched endovascular aortic repair
GDA: Gastroduodenal artery
IFU: Instructions for use
IRL: Infrarenal neck length
JAAA: Juxtarenal abdominal aortic aneurysm
MAE: Major adverse events
OSD: Off-the-shelf device
OSR: Open surgical repair
PET: Polyester
PMEG: Physician-modified endograft
PRAAA: Pararenal abdominal aortic aneurysm
SMA: Superior mesenteric artery
TAAA: Thoracoabdominal aortic aneurysm
TAMBE: Thoracoabdominal branch endoprosthesis
3D-CTA: Three-dimensional computed tomography angiography
